# Particle shape impacts export and fate in the ocean through interactions with the globally abundant appendicularian *Oikopleura dioica*

**DOI:** 10.1371/journal.pone.0183105

**Published:** 2017-08-30

**Authors:** Keats R. Conley, Kelly R. Sutherland

**Affiliations:** Department of Biology, Oregon Institute of Marine Biology, University of Oregon, Eugene, Oregon, United States of America; VIT University, INDIA

## Abstract

Marine microbes exhibit highly varied, often non-spherical shapes that have functional significance for essential processes, including nutrient acquisition and sinking rates. There is a surprising absence of data, however, on how cell shape affects grazing, which is crucial for predicting the fate of oceanic carbon. We used synthetic spherical and prolate spheroid microbeads to isolate the effect of particle length-to-width ratios on grazing and fate in the ocean. Here we show that the shape of microbe-sized particles affects predation by the appendicularian *Oikopleura dioica*, a globally abundant marine grazer. Using incubation experiments, we demonstrate that shape affects how particles are retained in the house and that the minimum particle diameter is the key variable determining how particles are ingested. High-speed videography revealed the mechanism behind these results: microbe-sized spheroids oriented with the long axis parallel to fluid streamlines, matching the speed and tortuosity of spheres of equivalent width. Our results suggest that the minimum particle diameter determines how elongated prey interact with the feeding-filters of appendicularians, which may help to explain the prevalence of ellipsoidal cells in the ocean, since a cell’s increased surface-to-volume ratio does not always increase predation. We provide the first evidence that grazing by appendicularians can cause non-uniform export of different shaped particles, thereby influencing particle fate.

## Introduction

**Fig 1:**
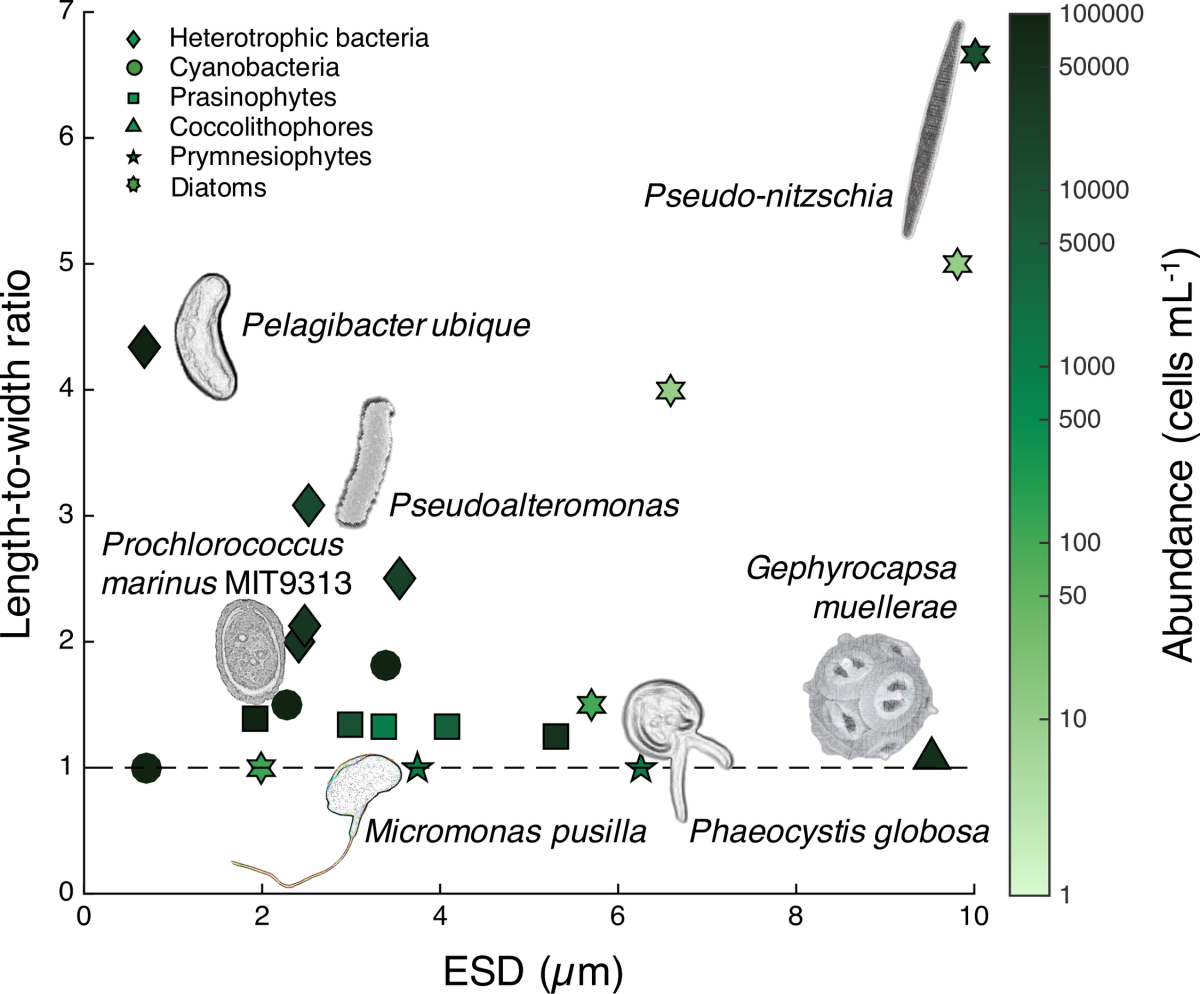
Morphologically diverse marine microbes. Length-to-width ratio versus surface-equivalent sphere diameter (ESD) [13] of some abundant free-living microbes in the upper ocean (S1 Table). Surface area (*S*) of ESD (${ESD=\frac{6}{\pi }S}^{1/2}$) was calculated based on the assumption of a prolate spheroid [13]. Color of points shows average abundances. Dashed black line represents the length: width ratio of a perfectly spherical cell.

The ocean is dominated by microorganisms that account for more than half of oceanic primary production and strongly mediate biogeochemical cycling [1]. Many of these marine microbes are non-spherical (Fig 1) [2–5]. For example, the cosmopolitan SAR11 bacterial clade accounts for a third of the total microbial community in the upper ocean, and the cells have a curved rod morphology [6]. *Prochlorococcus*, a cyanobacterium genus that contributes up to half of the photosynthetic biomass in certain oligotrophic regions, has differently shaped strains, ranging from spherical to oval (Fig 1) [7, 8]. Among prokaryotes, rod shape is more common than spherical [9]: a meta-analysis of the shapes of free-swimming bacterial genera found only 21% were spherical—the majority instead having rod-like shapes [10]. The pico- and nano-eukaryotes encompass a highly diverse group of organisms whose morphologies are also wide-ranging and often non-spherical [11, 12]. Their shapes may be further complicated by structural features such as spines, plates, or scales, and the formation of long filaments or chains.

Cell shape is central from an evolutionary perspective for both prokaryotes and eukaryotes. Rod- or filamentous-shaped bacterial cells are ancestral, while coccoid morphology is derived [14]. Cell morphology can exhibit rapid, phenotypic plasticity and may also be subject to selective pressures over longer evolutionary time scales. The selective forces influencing cell shape are varied [14]. Elongated shapes, for example, are advantageous for nutrient acquisition by increasing total surface area relative to volume for diffusion. Rod-like shape enables the establishment of poles and the sequestration of biochemicals such as attachment complexes, protein domains, and cell wall differentiation, and may facilitate adhesion in high-shear environments [14]. Eukaryotic phytoplankton are commonly rod-shaped since rods sink slower than spheres of equivalent volume, thereby promoting retention in the photic zone [11, 15]. Predation is certainly another important selective force on cell shape, however, our understanding of this interaction is much less complete [14].

Despite the morphological diversity of aquatic particles, the effect of particle size on predation has been more thoroughly studied than that of shape. Size-dependent predation has been extensively investigated using synthetic microspheres [16–22], which isolate the effect of size without the other conflating variables of live prey (e.g. surface properties, shape, density, motility). Non-spherical polymer micro-particles are presently not commercially available [23], and thus there is no comparable experimental method with which to test the potential for shape-based selection. Instead, the relationship between particle shape and predation has been primarily examined through the lens of predator-induced phenotypic plasticity [24, 25]. Predation often imposes a selection pressure for filamentous cells, small cells, and asymmetrical grazing-resistant morphologies [26], but shape remains a difficult variable to isolate and test experimentally.

Particle-grazer interactions in fluid are governed by hydrodynamics [4]. At microbial length scales, the hydrodynamics are low Reynolds number and dominated by viscous forces rather than inertial ones [27]. Rod-shaped particle behavior in a viscous fluid is known to be more complicated than that of spheres, characterized by tumbling behavior with varying orientations [28, 29]. Previous studies have also shown that particle shape has important ramifications for drag and the detachment of particles adhered to a surface [30]. The majority of models of marine particle behavior nonetheless assume spherical cell shape [3]. We hypothesized that the short axis of ellipsoidal particles should determine how they are captured by filter-feeders because theory predicts ellipsoidal particles in laminar flow tend to orient with the long axis parallel with fluid streamlines [29, 31], an orientation that minimizes drag [32].

We used the appendicularian *O*. *dioica* (Phylum: Chordata, Subphylum: Tunicata) as a model grazer because of the importance of appendicularians in ocean biogeochemical cycling. Appendicularians are planktonic herbivores whose abundance and individual grazing rates can equal or even exceed that of copepods [33, 34]. Appendicularians filter-feed by passing large volumes of water across an external mucous filter to consume microbe-sized particles down to four orders of magnitude smaller than themselves—encompassing the bacterial size range [35, 36]. The mucous filter, called the “house”, is periodically discarded and re-secreted at a rate of 2–40 houses day^-1^ [37]. Discarded appendicularian houses, containing concentrated, non-ingested prey, therefore constitute a major source of marine snow, typically contributing 28–39% of total particulate organic carbon export to the ocean’s interior [38].

**Fig 2:**
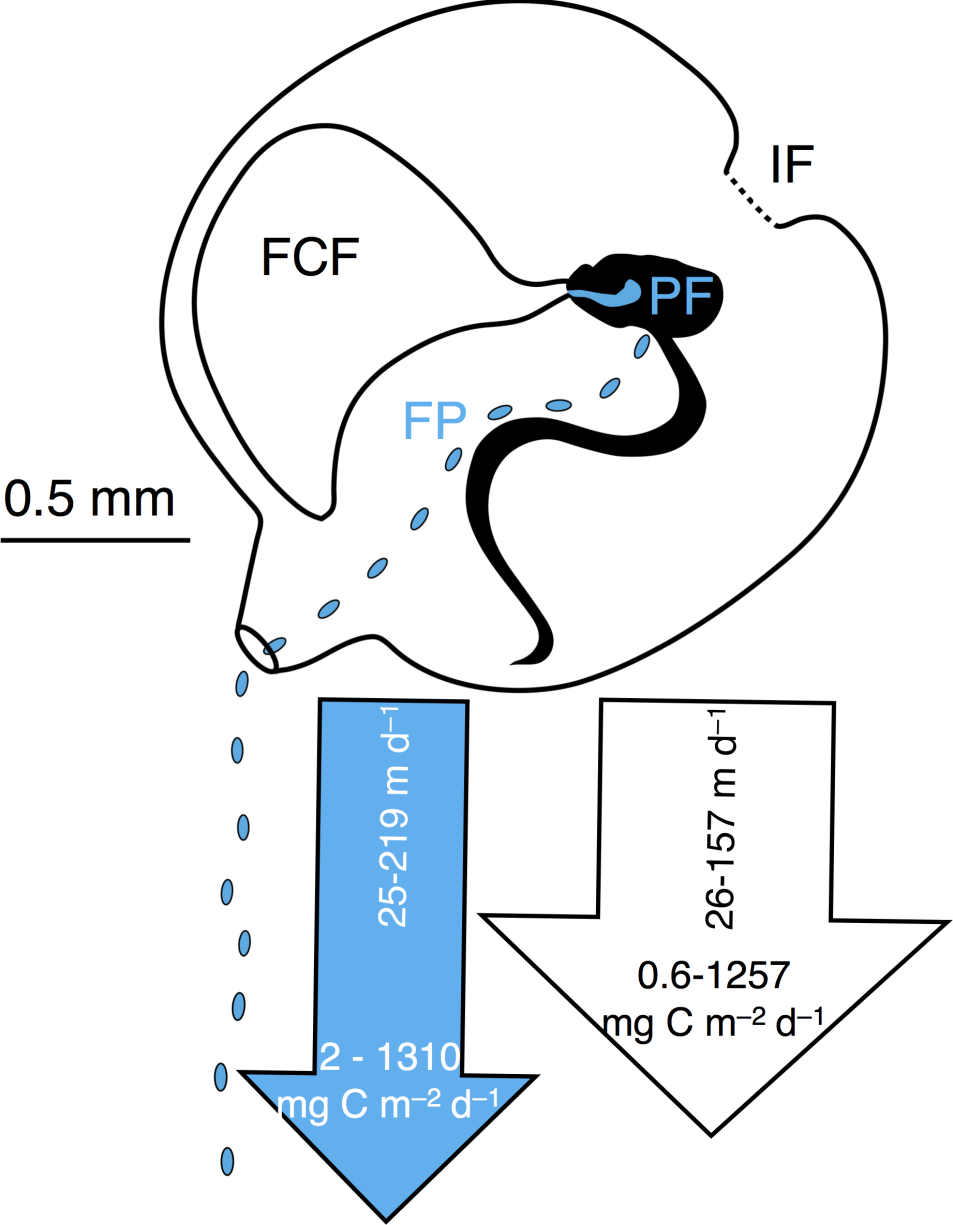
Grazing by *Oikopleura dioica* influences particle fate. Different fates of particles grazed by the appendicularian *Oikopleura dioica*: particles associated with the discarded house (white arrow) via retention on either the inlet filters (IF), food-concentrating filter (FCF), or house walls; particles captured on the pharyngeal filter (PF), ingested, and incorporated into fecal pellets (FP) (blue arrow). Arrow widths represent the average flux of houses (703 mg C m^-2^ d^-1^) and fecal pellets (446 mg C m^-2^ d^-1^) [44]. Arrow lengths represent the average sinking rates of houses (50 m day^-1^) ([38] and references therein) and fecal pellets (60 m day^-1^) [44]. Values show the range of flux for houses ([38] and references therein) and fecal pellets [42, 44], and the sinking rates of houses [42] and fecal pellets [44, 45]. Schematic of *O*. *dioica* by Jenna Valley.

We used incubation experiments coupled with high-speed videography of spheroidal and spherical microbead trajectories through the appendicularian house to determine how the axial ratio of particles affects grazing and fate. Appendicularians can differentially affect the fate of microbial prey through their complex feeding mechanisms, which result in particles either being retained in the external mucous house, or being ingested by the animal (Fig 2). Appendicularian grazing occurs through a series of distinct filtration steps. Prior to entering the house, particles are first screened by two coarse-meshed bilateral inlet filters that exclude large or spinous particles. Particles are then conveyed across the food-concentrating filter, which acts as a tangential filter to exclude water and concentrate particles [39]. The final filtration step occurs at the pharyngeal filter, which is suspended across the animal’s pharynx and collects particles for ingestion. Particles that adhere to the inlet or food-concentrating filters remain associated with the house (which is ultimately discarded), whereas particles captured by the pharyngeal filter are ingested and, depending on their digestibility, either incorporated into animal biomass or into fecal pellets (Fig 2). Dense fecal pellets from *O*. *dioica* tend to be expelled from the house and thus represent a separate pathway for vertical flux [40] sinking up to 200 m day^-1^ [41] (Fig 2). In some areas, *O*. *dioica* has been observed to be the second-largest contributor to total fecal carbon flux of any individual species [42, 43]. Appendicularian grazing can therefore profoundly alter planktonic particle diversity and fate, both within and below the upper mixed layer.

Because of the ubiquity of rod-shaped microbes in the ocean (Fig 1), the scarcity of data on how shape affects the fate of particles in the ocean represents a notable gap. We show that particle interactions with the appendicularian feeding-filters depends on particle shape and also particle size, and that, irrespective of size, spheroidal particles are retained in the house or captured by the pharyngeal filter like spheres of equivalent width. We propose that ellipsoidal cells may in some cases benefit from an increased surface-to-volume ratio without necessarily incurring an increased cost of predation, since grazing by *O*. *dioica* appears based upon minimum cell width.

## Materials and methods

### Incubation experiments

**Fig 3:**
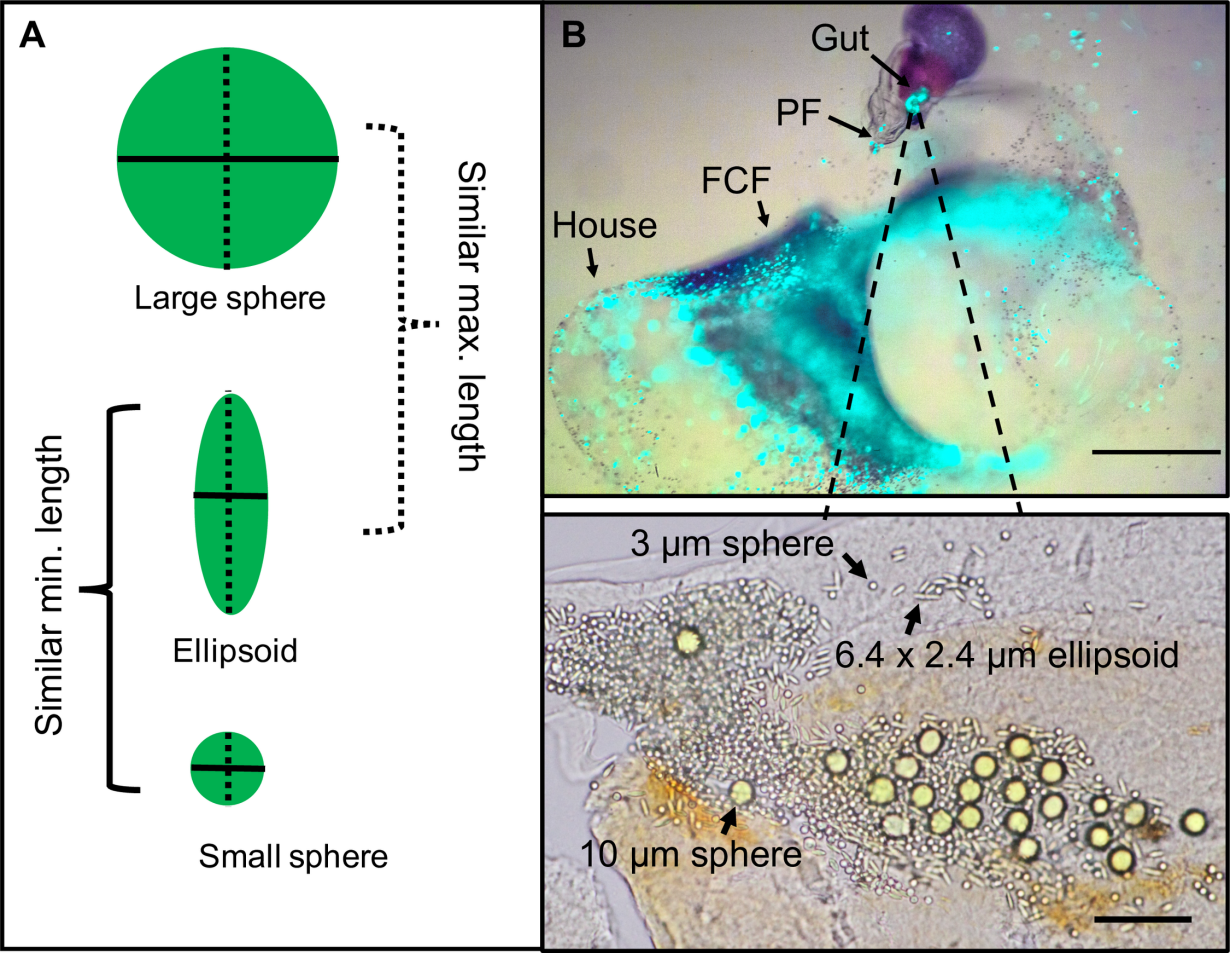
Experimental bead mixture for incubations. (A) Schematic of experimental bead mixture used in each of three incubation experiments with *Oikopleura dioica*. (B) Top: *O*. *dioica* in house filtering a mixture of *Rhinomonas reticulata* (red, ~17 μm diameter) and fluorescent 10 μm microspheres (green). PF: pharyngeal filter; FCF: food-concentrating filter. Scale bar 0.5 mm. Bottom: experimental bead mixture (3–10 μm) in the gut post-incubation. Scale bar 50 μm.

We tested a mix of three polystyrene bead types (small spheres, prolate spheroids, and large spheres) using three separate grazing incubation experiments (Table 1). Spheroidal particles were synthesized using a toluene-stretching technique [23]. To account for the possible effect of the stretching procedure on bead surface properties, we prepared control spherical microbeads using the same polyvinyl film embedment procedure but without mechanically stretching the film. The spheroids had one axis similar to the diameter of the small spheres, and one axis similar to the diameter of the larger spheres (Fig 3A). The large spheres were always slightly larger than the maximum dimension of the spheroids due to constraints of the commercial availability of different sizes of microspheres (Fig 3A, Table 1). In each incubation experiment, all three bead types were applied simultaneously. Particle concentrations were selected based on the concentration of similarly-sized cells in the upper ocean [17] (Table 1).

**Table 1:** Experimental conditions for three incubation experiments.

Incubation experiment	Bead mixture	Diameter (μm)	Volume (μm^3^)	Emission (nm)	T_0_ conc. (mean beads mL^-1^ ± S.D.)	T_f_ conc. (mean beads mL^-1^ ± S.D.)	Control T_0_ conc. (mean beads mL^-1^ ± S.D.)	Control T_f_ conc. (mean beads mL^-1^ ± S.D.)
1	Small sphere	0.32	0.14	660	8.1 x 10^5^ ± 5.9 x 10^5^	1.0 x 10^6^ ± 6.2 x 10^5^	5.0 x 10^5^ ± 2.8 x 10^5^	6.0 x 10^5^ ± 1.4 x 10^5^
	Prolate spheroid	0.3 x 0.7	0.14	441	1.1 x 10^5^ ± 7.1 x 10^4^	1.3 x 10^5^ ± 1.0 x 10^5^	1.8 x 10^5^ ± 3.5 x 10^4^	1.0 x 10^5^ ± 7.0 x 10^4^
	Large sphere	1	4.2	441	1.8 x 10^5^ ±1.8 x 10^5^	7.8 x 10^4^ ± 6.2 x 10^4^	1.8 x 10^5^ ± 3.5 x 10^4^	1.3 x 10^5^ ± 1.8 x 10^5^
2	Small sphere	0.5	0.065	763	2.6 x 10^6^ ± 6.8 x 10^5^	2.6 x 10^6^ ± 8.4 x 10^5^	2.9 x 10^6^ ± 5.0 x 10^5^	2.5 x 10^6^ ± 6.0 x 10^5^
	Prolate spheroid	0.5 x 1.4	0.065	441	1.7 x 106 ± 5.5 x 10^5^	1.6 x 10^6^ ± 4.5 x 10^5^	1.5 x 10^6^ ± 3.2 x 10^5^	1.9 x 10^6^ ± 2.8 x 10^5^
	Large sphere	1.75	22.5	441	1.4 x 10^6^ ± 6.4 x 10^5^	1.2 x 10^6^ ± 4.6 x 10^5^	1.0 x 10^6^ ± 5.8 x 10^4^	1.5 x 10^6^ ± 3.3 x 10^5^
3	Small sphere	3	14	529	2.1 x 10^5^ ± 6.2 x 10^4^	2.1 x 10^5^ ± 5.8 x 10^4^	2.6 x 10^5^ ± 6.1 x 10^4^	2.1 x 10^5^ ± 2.0 x 10^4^
	Prolate spheroid	2.4 x 6.4	14	441	2.6 x 10^5^ ± 8.9 x 10^4^	2.4 x 10^5^ ± 6.6 x 10^4^	3.0 x 10^5^ ± 4.3 x 10^4^	3.1 x 10^5^ ± 5.9 x 10^4^
	Large sphere	10	524	441	6.4 x 10^4^ ± 2.3 x 10^4^	5.7 x 10^4^ ± 2.1 x 10^4^	7.8 x 10^4^ ± 3.4 x 10^4^	5.9 x 10^4^ ± 6.4 x 10^3^

All experimental animals were obtained from the appendicularian culture facility at the Sars Centre for Marine Molecular Biology in Bergen, Norway. For each incubation, either late day-5 or early day-6 animals were used to ensure consistent animal size (Table 2). Animals were transferred from 1-μm filtered seawater (FSW) to a 0.2-μm FSW bath and probed to abandon their houses using a wide-bore pipette. We then transferred animals into a second 0.2-μm FSW bath in order to allow them to build a new, clean house prior to use in the experiment. Incubation chambers (44 mL) were pre-rinsed with 0.2-μm FSW and filled with 0.2-μm FSW. Experimental bead mixtures (small spheres, prolate spheroids, and large spheres) were diluted with seawater, and then pipetted into the incubation chamber. The chambers were gently inverted several times to homogenize the beads prior to the addition of an animal. One animal was randomly assigned to each incubation container, and two or three chambers with no animal (i.e., only experimental particles) served as controls. All incubations were performed on a lab bench at 20°C. Since we sought to compare the relative proportion of different bead types in the house and guts, all incubations were 10 min in duration, which is the approximate gut clearance time for *O*. *dioica* [46]. *O*. *dioica* has a maximum *in situ* filtration rate of 12.5 ml animal^-1^ hr^-1^ [34], so the volume of the incubation container allowed for a relatively constant ambient particle field (~5% of the chamber volume filtered). Each chamber was sampled for 1 mL of the initial water prior to the addition of an animal (T_0_) and the water at the end of the incubation (T_final_). At the conclusion of the incubation, we recorded whether each animal was still in the house. We then carefully pipetted the animal and house using a wide-bore pipette and transferred them to a glass embryo dish with 0.2-μm FSW. If animals were still filtering, they were probed to abandon their house, photographed for size measurements, and then the whole animal was collected using a Pasteur pipette for subsequent gut content analysis. Houses were also collected with a micropipette set to a fixed volume (300 μL). All samples were collected into 1.8 mL cryotubes, promptly fixed with 0.1% (by volume) of 25% gluteraldehyde, and refrigerated until analysis.

**Table 2:** Observational results from incubation experiments with *Oikopleura dioica*.

Experimental observations	3–10 μm	0.5–1.75 μm	0.3–1.0 μm
*O*. *dioica* trunk length, mm (mean ± SD)	0.79 ± 0.12 (*n* = 12)	0.79 ± 0.10 (*n* = 12)	1.0 ± 0.18 (*n* = 10)
Proportion of animals filtering at T_final_	33.3%	100.0%	66.7%
Proportion of animals with beads in gut	50.0%	58.3%	55.6%
Proportion of animals with >10 beads in gut	33.3%	58.3%	44.4%
Total number of beads in gut (mean ± SD)	195 ± 262	266 ± 160	141 ± 111

T_final_ is the water sampled at the end of the 10-minute incubations.

Beads were quantified using a Nikon Eclipse Ei compound microscope. Beads in the 3–10 μm size range were analyzed using epifluorescence microscopy with a 10x objective. Concentrations of T_0_ and T_final_ samples for 3–10 μm beads were determined using a Reichert Bright-Line hemacytometer (0.1 mm deep, CAT # 1492). Concentration measurements for beads <3 μm were made using a Petroff-Hausser Bacteria Counter (0.2 mm deep, Fisher Scientific CAT # 02-671-13) with a 20x or 40x objective using dark-field light microscopy [47]. All samples were vortexed prior to counting and a sufficient number of grids were counted to achieve an average total bead count of ~100 (20 μL per sample).

Gut samples were prepared by directly mounting animals onto a glass slide and treating them with 10 μl recombinant PCR Grade Proteinase K solution from *Pichia pastoris* (Sigma-Aldrich, Cat No. 3115887001) to degrade the tissue for better differentiation of beads (Fig 3B). Gut samples were analyzed using a 40x objective and photographed using a Canon EOS 5D. We performed manual particle counting using the Multi-point Tool in Image J [48]. Houses from the 3–10 μm were intact enough to mount on a microscope slide and perform particle counts identically to the guts. Houses from the other incubations had dissolved and thus were counted identically to the water samples using a Petroff-Hausser chamber. Data analysis for gut samples was restricted to those animals with >10 beads. Because of the inherent fragility of house and gut samples, all parameters could not be measured for all samples.

### Videography

Immature day-6 *O*. *dioica* were filmed individually using a Sony 4K FDR-AX100 (HD resolution, 120 fps) mounted to a Nikon Eclipse E400 with a 10x objective using a Martin Microscope M99 Camcorder Adapter. Animals were filmed in a glass embryo dish filtering a suspension of 10 μm-diameter spheres and spheroids of similar width (L = 22 ± 2.4 μm and W = 7.8 ± 1.1 μm; *n* = 5; mean ± SD). Videos were converted to image stacks for velocity and trajectory analysis in ImageJ [48]. Particle tracking velocimetry was performed by manually tracking individual particles between frames using the plugin MTrackJ [49]. Particle coordinates were used to calculate velocities and net-to-gross displacement ratios (NGDR) for spherical and spheroidal particles. All trajectories were obtained from one day-6 individual. Trajectories for net-to-gross displacement were restricted to distances >100 μm.

For measurements of spheroidal particle orientation, the image stack was registered using the StackReg plugin with an Affine transform to account for the inflation and deflation of the feeding mesh. Images were then inverted and frames for trajectory analysis were color-coded in one of six channels using the Stack-to-Hyperstack tool. Stacks were Z-projected and the composite image was used to show trajectories. Measurements of spheroidal particle orientation were made using the straight line angle tool in ImageJ [48] and converted to be relative to the fluid flow.

### Statistics

Selection for the different shaped beads was calculated using the Chesson’s selectivity index [50]:

$$\alpha_{i}=\frac{d_{i}/e_{i}}{\sum_{j=1}^{k}\left(\frac{d_{j}}{e_{j}}\right)}$$

where *i* = 1, 2, ….*k*

*k* is the number of prey categories, *d* is the proportion of prey type *i* in the diet, *e* is the proportion of prey type *i* in the environment. The selectivity coefficient *α*_*i*_, which is independent of the relative abundance of different prey types, ranges from 0 to 1 [50]. Random feeding is defined by $\alpha_{i}=\frac{1}{k}$, with values <*α*_*i*_ and >*α*_*i*_ indicating negative and positive selection, respectively. In this study, $\frac{1}{k}=0.333$ since each incubation tested three bead types. The null hypothesis of no selection $\left(\alpha_{i}=\frac{1}{k}\right)$ was tested using t-tests. Data were arcsine-square root transformed to meet the assumption of normality and a Bonnferoni correction of alpha level (0.05/number of t-tests) was used to provide an overall error rate of 0.05 [51].

## Results

**Fig 4:**
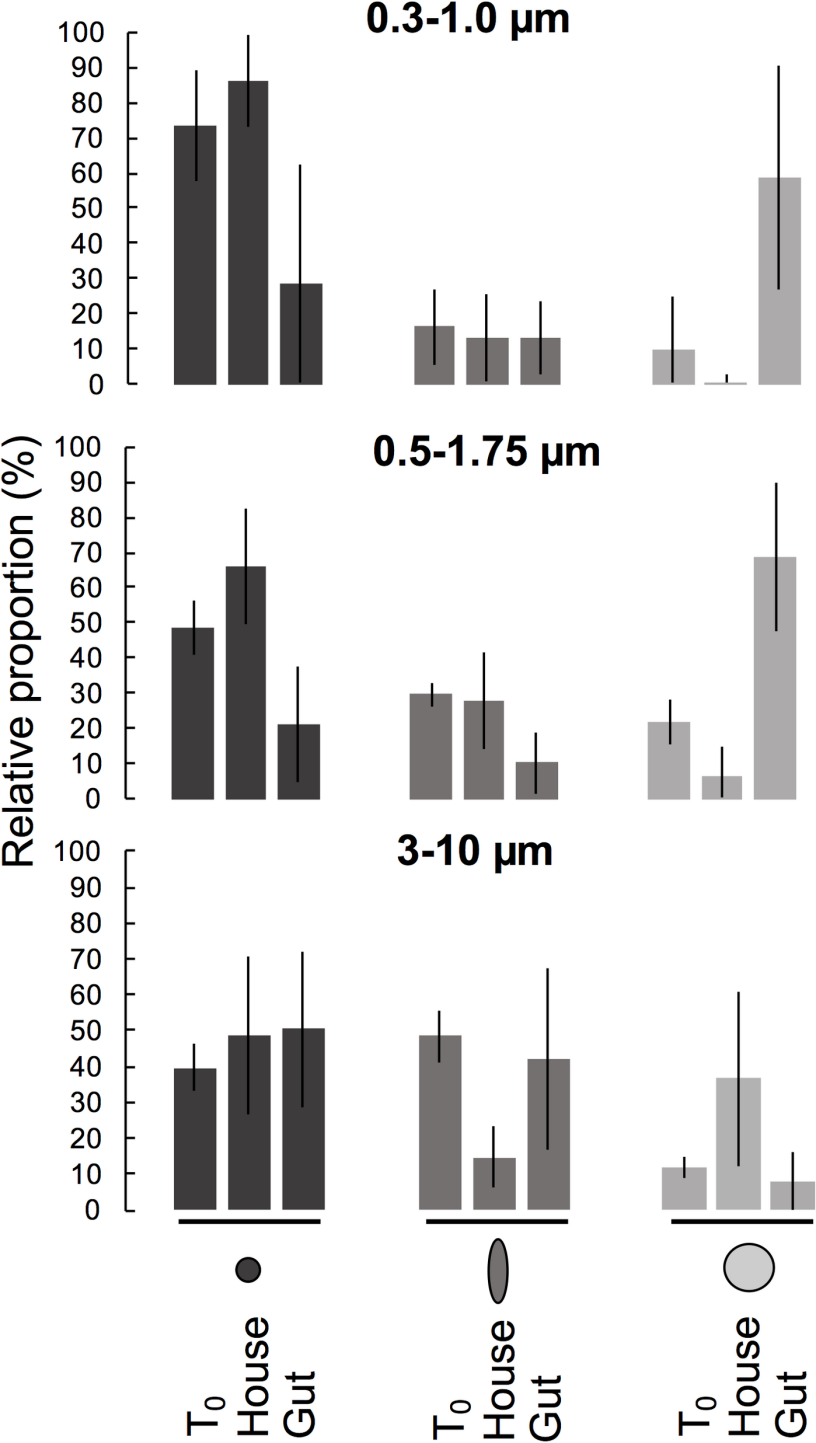
Fate of different shaped beads from three incubation experiments. Relative proportions (mean ± SD) of various bead mixes in the ambient water at the start of the incubation (T_0_), gut, and house of the appendicularian *Oikopleura dioica*.

**Fig 5:**
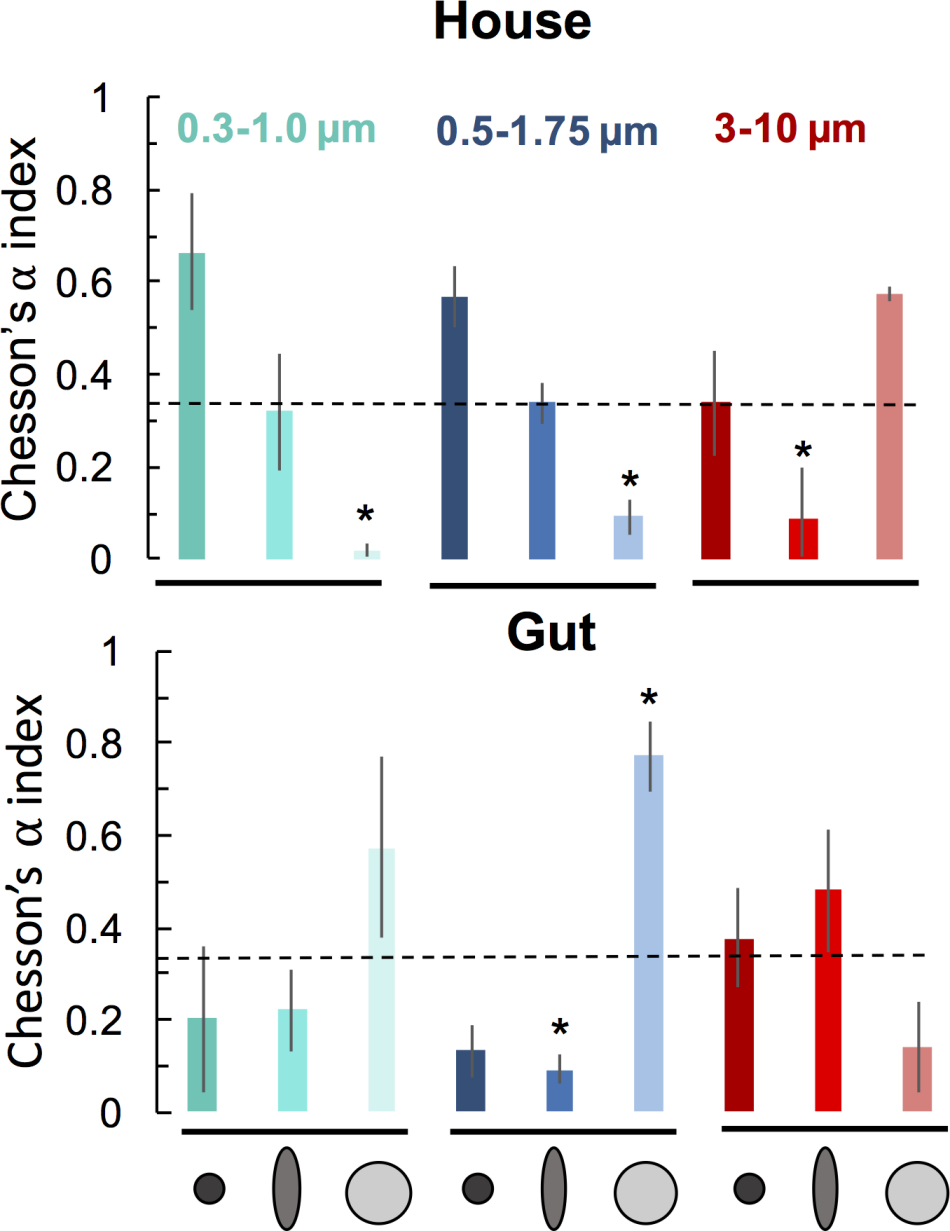
Particle shape affects selection by the appendicularian *Oikopleura dioica*. Selectivity coefficients (mean Chesson’s α-index ± SE) for different bead types in the houses and guts from each of three incubation experiments with *Oikopleura dioica*. * indicates selectivity values that were significantly different from non-selectivity (α = 0.33, dashed line) tested using t-tests with a Bonnferoni correction of alpha level (p ≤ 0.0028) (Table 3).

Three separate incubation experiments with *O*. *dioica* grazing on environmentally relevant particle sizes (0.3–10 μm) showed that shape differentially affected particle retention patterns in the house and gut (Fig 4). Retention patterns in house were more variable than that in the gut and interrelated with particle size. Regardless of size, spheroids were always ingested according to their minimum diameter (Figs 4 and 5). The mean Chesson’s α-index for spheroids was always more similar to that of spheres of similar minimum diameter than to spheres of similar maximum diameter (Fig 5), indicating that the minimum dimension of the spheroid appears to determine ingestion rates.

In addition to particle shape, size also had a pronounced effect on particle fate. The relative proportion of different bead types were consistently inverse in the house and in the guts (Fig 4). In the 3–10 μm incubation, the 10 μm spheres had a positive selectivity index in the house, while the 2.4 x 6.4 μm spheroids had a negative selectivity index, and 3 μm spheres had neutral selectively (Fig 5; Table 3). The reverse pattern occurred for both the 0.5–1.75 μm incubation and the 0.3–1.0 μm incubation: large spheres were disproportionately ingested while small spheres and spheroids predominated in the house (Fig 4). In the houses from the two incubations with smaller-sized beads (0.3–1.75 μm), ellipsoidal beads had a mean Chesson’s α-index in between that of the small and large spheres, indicating intermediate, but non-selective retention. Absolute counts of beads in guts, along with the change in relative proportions of beads from T_0_ suggest a sharp reduction in ingestion of particles below ~1.0 μm (Fig 4). The proportion of animals filtering at T_final_ was lowest for the incubation with the largest size particle assemblage (3–10 μm), and the animals in this experiment also had the lowest proportion of animals with >10 beads in the gut (Table 2), implying reduced efficacy of feeding with this particle regime. We therefore infer that while *O*. *dioica* houses may affect a broader range of particle sizes, the animals themselves are restricted to ingesting a size range of 1.0 μm to <10 μm. Both particle shape and size therefore affect predation by appendicularians and influence the ultimate fate of the particles.

**Table 3:** Statistical results for incubation experiments.

	House			Gut		
Bead size	*t*-statistic	df	p-value	*t*-statistic	df	p-value
0.3	2.4725	6	0.0483*	-1.1696	3	0.327
0.3 x 0.7	-0.72759	6	0.494	-1.3777	3	0.262
1	-11.179	6	<0.001**	1.1402	3	0.337
0.5	3.4275	9	0.00754*	-3.6531	6	0.0107*
0.5 x 1.4	-0.047972	9	0.963	-5.385	6	0.002**
1.75	-5.1398	9	<0.001**	5.2286	6	0.002**
3	-0.19261	8	0.852	0.001788	5	0.999
2.4 x 6.4	-7.8573	8	<0.001**	-1.0277	5	0.351
10	2.9004	8	0.0199*	-2.4019	5	0.0615

Statistical comparisons are from T-Tests on the selectivity coefficient *α*_*i*_ values versus the null hypothesis of no selection $\left(\alpha_{i}=\frac{1}{k}\right)$.

*p ≤ 0.05

**p ≤ 0.0028 (Bonnferoni correction).

High-speed micro-videography of the food-concentrating filter revealed the mechanism behind the similar retention of spheroids and spheres of equivalent width: no significant difference was observed between the net-to-gross displacement ratio (NGDR) of 8 x 22 μm spheroids and 10 μm spheres (ANOVA, F_1,54_ = 0.457, *P* = 0.502, *n* = 26 and *n* = 29 for spheres and spheroids, respectively), nor was there a significant difference in their speeds through the filter (two-factor ANOVA, F_1,34_ = 2.133, *P* = 0.153, *n* = 31 for both spheres and spheroids). The kinematics of spheroidal particles were thus similar to spheres of equivalent width, consistent with results from the incubation experiments.

**Fig 6:**
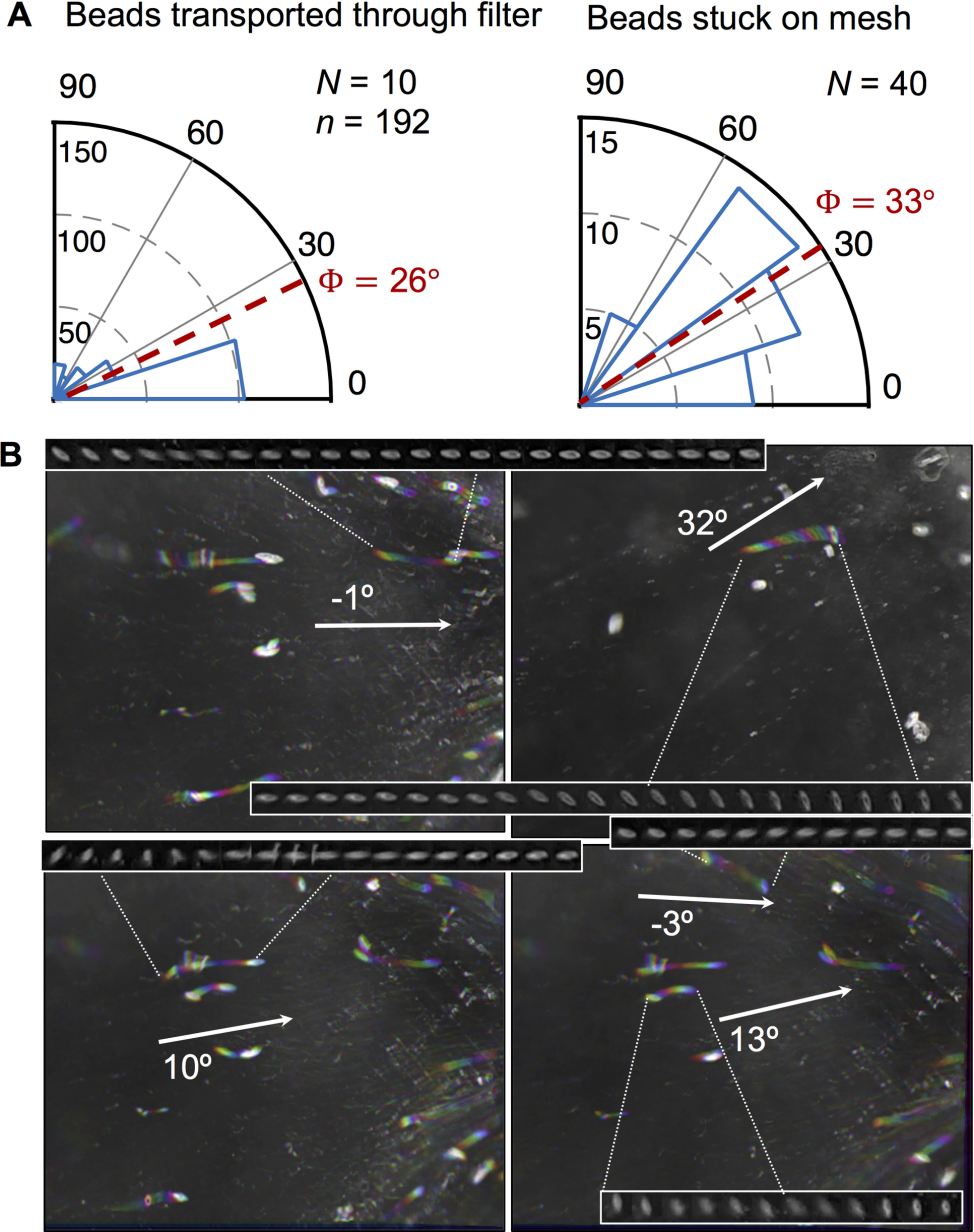
Trajectories and orientation of spheroidal microbeads through the feeding-filter of *Oikopleura dioica*. (A) Frequency histograms show the angles for prolate spheroid microbeads (7.8 x 22 μm) suspended in the fluid of the food-concentrating filter and adhered to the filter mesh of *Oikopleura dioica*. Sectors correspond to number of beads observed, Ф is the grand mean direction for all beads, *N* is the number of independently measured beads, *n* is the instantaneous angles pooled for all beads. Angle measurements for beads adhered to the mesh represent independent measurements for *N* = 40 beads, whereas measurements for beads suspended in the fluid represent instantaneous angles pooled for all individuals, *n* = 192, from the trajectories of *N* = 10 beads. All angles are relative to the fluid flow. (B) Five sample trajectories of beads transported through the food-concentrating filter (S1 Video). Frames were colored-coded (red, yellow, green, cyan, blue, magenta) so that the color order shows direction and white indicates a particle has not moved. Arrows show directions of fluid flow. Montages show the bead orientations for the respective trajectory.

Spheroids tended to passively orient short-end forward when they moved through the low Reynolds number regime of the food-concentrating filter (Fig 6) and were more variably oriented when adhered to the filter mesh (Fig 6). Beads suspended in the fluid had a lower mean orientation angle (Ф = 26°), indicating greater alignment with the fluid flow than beads stuck to the mesh (Ф = 33°) (Fig 6). Spheroidal particles exhibited a tumbling behavior, changing orientation as they were conveyed through the filter (Fig 6B).

## Discussion

The upper ocean is dominated by non-spherical particles (Fig 1), which are central in biogeochemical cycling. Our findings demonstrate that the shape of microbe-sized particles affects their fate through their interactions with the filtration apparatus of a globally abundant marine grazer, *O*. *dioica*. The results from our incubation experiments show that, regardless of the particle size, the minimum particle diameter is the key variable affecting how particles are grazed (Figs 4 and 5). This study represents the first experimental evidence of differential grazing based solely on particle shape and implies grazing by appendicularians can cause non-uniform export of different shaped particles.

Although feeding structures may vary widely [52], encounter between a prey particle and a food-collecting element is a somewhat generalizable process because of the finite number of hydrodynamic particle encounter mechanisms [53, 54]. The appendicularian pharyngeal filter can capture particles smaller than the mesh pores through direct interception and diffusional deposition of particles onto the sticky filter fibers [35]. These small-scale interactions between a particle and filter fiber are applicable to other planktonic grazers. Phagotrophic protists, dominant bacterivores in aquatic systems, exhibit a wide variety of feeding strategies, but food acquisition depends initially on prey contact and capture by a food-collecting element prior to ingestion [55]. Copepods capture prey using bristled appendages at Reynolds numbers ~10^−2^ to 10^−1^ [56]. Previous work has shown that elongated diatoms in the siphon flow of a copepod’s feeding appendages tend to orient with their long axes parallel to streamlines [57], corroborating our high-speed videography observations that prolate spheroids in the laminar flow regime of the appendicularian food-concentrating filter tend to passively orient short-end forward (Fig 4). Mathematical predictions and empirical evidence also support that spheroidal particles at low Reynolds numbers preferentially align with fluid streamlines, periodically flipping orientation depending on the hydrodynamic shear [29, 31, 58]. The kinematics and differential retention of spheroidal particles by appendicularians may therefore have broader applicability to other pelagic and benthic bacterivores that rely on different feeding appendages to capture prey.

The few studies that have examined the ramifications of microbe shape for predation [26, 57, 59–61] are subject to the methodological limitations of live prey—namely, the possible confounding effects of surface properties, motility, and morphological variability (e.g. flagella, pili, fibrils, gel matrices, coccoliths or protist scales). Ours is the first study to isolate the effect of particle shape on differential grazing using synthetic particles of uniform dimensions, densities, and surface properties. Our findings demonstrate particle length-to-width ratios influence particle fate—specifically, whether particles were ingested or remained adhered to the appendicularian house (Fig 2 and Fig 4). Particle shape can therefore affect the composition of marine snow aggregates produced by mucous filter-feeders.

Non-uniform selection has implications for particle removal from the upper ocean and for carbon export to the ocean’s interior. Appendicularians are a major contributor to marine snow in the euphotic and mesopelagic zones through the production of discarded houses and fecal pellets [62, 63]. Although houses and fecal pellets are aggregations of small particles, particles in these forms are subject to different fates [41, 42] (Fig 2). The carbon content of discarded houses varies by species and is also affected by retained particles, ranging from 2.6 to 56 ug C house^-1^ with flux rates spanning 0.6 to 1257 mg C m^-2^ d^-1^ [38]. An individual *Oikopleura* can produce over 300 fecal pellets per day [64], with carbon content ranging from 3.55 x 10^−14^ to 1.18 × 10^−11^ μg C fecal pellet^-1^ [65] and flux rates of 2 to 1310 mg C m^-2^ d^-1^ [42, 44]. Houses and fecal pellets are also subject to different sinking rates (Fig 2) and microbial mineralization may occur during their descent. Our results suggest that large (≥10 μm diameter) and submicron particles are more likely to remain associated with the appendicularian house, which generally contain more labile carbon than fecal pellets [67] and contributes to greater carbon flux rates (Fig 2), whereas micron sized particles (≥10 μm diameter) are more likely to either be assimilated into biomass or compacted into fecal pellets that sink at faster rates than the houses (Fig 2). These findings therefore have ramifications for the microbial loop [67], indicating that cell size and shape may influence which cells are recycled into the food chain via the microbial loop versus exported from the surface ocean.

We found that particle size also strongly influences selection and particle fate, which is consistent with previous studies [16, 18, 35]. Appendicularians efficiently ingested particles in the intermediate size range (1–3 μm), leaving a higher-than-ambient proportion of large particles (10 μm) and submicron particles (<0.5 μm) in the house. Previous authors have similarly shown reduced ingestion of particles below ~1 μm [16, 18]. Since size and shape are interrelated metrics, our results extend the current understanding of size-based selection by identifying how length-to-width ratios affect retention efficiencies. It is well-established that a cell’s length-to-width ratio is of fundamental importance for a variety of physical and physiological processes, including photosynthesis, diffusion, active motility and passive dispersal [14]. Our results suggest that the predation risk experienced by ellipsoidal cells is similar to the predation risk of spherical cells with a diameter equivalent to the minimum diameter of the spheroid. This identifies an additional, potential explanation for the prevalence of ellipsoidal cells in the ocean.

## Supporting information

S1 Table Published size and abundance measurements of marine microbes included in Fig 1B. (DOCX)

S1 Video Prolate spheroid microbeads (22 x 7.8 μm) transported through the food-concentrating filter of *Oikopleura dioica*. (AVI)

S1 Dataset Count data from incubation experiments used for Figs 3 and 4. (XLSX)

S2 Dataset Spheroidal microbead trajectory data used for Fig 5. (XLSX)
