# Supplementary material for: Particle shape impacts export and fate in the ocean through interactions with the globally abundant appendicularian Oikopleura dioica
Source: PLoS One. 2017 Aug 30;12(8):e0183105. doi: 10.1371/journal.pone.0183105 (PMC5576645; doi:10.1371/journal.pone.0183105)
Supplement: S1 Table — (DOCX) [file pone.0183105.s001.docx]

| **Category** | **Genus, species** | **Mean width**  (μm) | **Mean length** (μm) | **Source for size measurements** | **Abundance**  (cells mL^-1^) | **Source for abundance** |
| --- | --- | --- | --- | --- | --- | --- |
| Heterotrophic bacteria | *Pelagibacter ubique* | 0.15 | 0.65 | 69 | 3.45 x 10^5^ | 70 |
| Heterotrophic bacteria | *Roseobacter denitrificans* | 0.75 | 1.5 | 71 | 3.5 x 10^4^ | 72 |
| Heterotrophic bacteria | *Roseobacter litoralis* | 0.75 | 1.6 | 71 | 3.5 x 10^4^ | 72 |
| Heterotrophic bacteria | *Vibrio* spp. | 0.65 | 2 | 73 | 1.3 x 10^4^ | 72 |
| Heterotrophic bacteria | *Pseudoalteromonas* | 1 | 2.5 | 73 | 2.1 x 10^4^ | 72 |
| Cyanobacteria | *Prochlorococcus marinus* MIT9313 | 0.8 | 1.2 | 7 | 1 x 10^5^ | 8 |
| Cyanobacteria | *Prochlorococcus marinus* MED4 | 0.7 | 0.7 | 7 | 1 x 10^5^ | 8 |
| Cyanobacteria | *Synechococcus* | 1.1 | 2 | 74 | 1.9 x 10^5^ | 74 |
| Prasinophytes | *Micromonas pusilla* | 1.1 | 1.5 | 75 | 1 x 10^4^ | 76 |
| Prasinophytes | *Ostreococcus tauri* | 0.7 | 1 | 77 | 1.0 x 10^5^ | 76 |
| Prasinophytes | *Bathycoccus prasinos* | 1.5 | 2 | 78 | 3.8 x 10^3^ | 79 |
| Prasinophytes | *Pycnococcus provasolii* | 3.4 | 3.4 | 80 | 1 x 10^3^ | 81 |
| Prasinophytes | *Prasinoderma coloniale* | 2 | 2.5 | 82 | 1 x 10^3^ | 76 |
| Coccolithophores | *Gephyrocapsa muellerae* | 3.8 | 4.1 | 83 | 1 x 10^4^ | 83 |
| Prymnesiophytes | *Phaeocystis pouchetii* | 6.25 | 6.25 | 84 | 1 x 10^3^ | 85 |
| Prymnesiophytes | *Phaeocystis globosa* | 3.75 | 3.75 | 84 | 1 x 10^3^ | 85 |
| Diatoms | *Pseudo-nitzschia brasiliana* small | 1.8 | 12 | 86 | 1 x 10^4^ | 86 |
| Diatoms | *Skeletonema costatum* | 2 | 3 | 5 | 1 x 10^2^ | 5 |
| Diatoms | *Thalassionema nitzschioides* small | 2 | 10 | 5 | 1 x 10^1^ | 5 |
| Diatoms | *Nitzschia* sp. small | 1.5 | 6 | 5 | 1 x 10^1^ | 5 |
| Diatoms | *Chaetoceros* spp*. pequeñas* | 2 | 2 | 5 | 1.2 x 10^2^ | 5 |

**References**

69. Munn C. Marine microbiology. 2^nd^ ed. New York: Garland Science; 2011.

70. Morris RM, Rappé MS, Connon SA, Vergin KL, Siebold WA, Carlson CA, et al. SAR11 clade dominates ocean surface bacterioplankton communities. Nature. 2002; 420(6917): 806-10.

71. Shiba T. *Roseobacter litoralis* gen. nov., sp. nov., and *Roseobacter denitrificans* sp. nov., aerobic pink-pigmented bacteria which contain bacteriochlorophyll a. Syst Applied Microbiol. 1991; 14(2): 140-5.

72. Wietz M, Gram L, Jørgensen B, Schramm A. Latitudinal patterns in the abundance of major marine bacterioplankton groups. Aquat Microb Ecol. 2010; 61(2): 179-89.

73. Vos P, Garrity G, Jones D, Krieg NR, Ludwig W, Rainey FA, et al. Bergey's Manual of Systematic Bacteriology: Volume 3: The Firmicutes. Berlin: Springer Science & Business Media; 2011.

74. Waterbury JB, Watson SW, Guillard RR, Brand LE. Widespread occurrence of a unicellular, marine, planktonic, cyanobacterium. Nature. 1979; 277(5694): 293-4.

75. Booth BC, Lewin J, Norris RE. Nanoplankton species predominant in the subarctic Pacific in May and June 1978. Deep Sea Res A. 1982; 29(2): 185-200.

76. Vaulot D, Eikrem W, Viprey M, Moreau H. The diversity of small eukaryotic phytoplankton (≤ 3 μm) in marine ecosystems. FEMS Microbiol Rev. 2008; 32(5): 795-820.

77. Chrétiennot-Dinet M, Courties C, Vaquer A, Neveux J, Claustre H, Lautier J, et al. A new marine picoeucaryote: *Ostreococcus tauri* gen. et sp. nov.(Chlorophyta, Prasinophyceae). Phycologia. 1995; 34(4): 285-92.

78. Eikrem W, Throndsen J. The ultrastructure of *Bathycoccus* gen. nov. and *B. prasinos* sp. nov., a non-motile picoplanktonic alga (Chlorophyta, Prasinophyceae) from the Mediterranean and Atlantic. Phycologia. 1990; 29(3): 344-50.

79. Collado-Fabbri S, Vaulot D, Ulloa O. Structure and seasonal dynamics of the eukaryotic picophytoplankton community in a wind‐driven coastal upwelling ecosystem. Limnol Oceanogr 2011; 56(6): 2334-46.

80. Guillard RR, Keller MD, O'Kelly CJ, Floyd GL. *Pycnococcus provasolii* gen. et sp. nov., a coccoid prasinoxanthin‐containing phytoplankter from the western north Atlantic and Gulf of Mexico. J Phycol. 1991; 27(1): 39-47.

81. Zingone A, Sarno D, Siano R, Marino D. The importance and distinctiveness of small-sized phytoplankton in the Magellan Straits. Polar Biol. 2011; 34(9): 1269-84.

82. Hasegawa T, Miyashita H, Kawachi M, Ikemoto H, Kurano N, Miyachi S, et al. *Prasinoderma coloniale* gen. et sp. nov., a new pelagic coccoid prasinophyte from the western Pacific ocean. Phycologia. 1996; 35(2): 170-6.

83. Winter A, Siesser WG. Coccolithophores: Cambridge University Press; 2006.

84. Medlin L, Zingone A. A taxonomic review of the genus *Phaeocystis*. Biogeochemistry. 2007; 83(1-3): 3-18.

85. Vogt M, O'Brien C, Peloquin J, Schoemann V, Breton E, Estrada M, et al. Global marine plankton functional type biomass distributions: *Phaeocystis* spp. Earth Syst Sci Data. 2012; 4(1):107-20.

86. Villac MC, Melo S, Menzes M, Tenenbaum DR. *Pseudo-nitzschia brasiliana* (Bacillariophyceae), an opportunistic diatom on the coast of the state of Rio de Janeiro, Brazil. Atlântica, Rio Grande. 2005; 27(2):139-45.
